# Supplementary material for: Effects of Lactobacillus rhamnosus and Enterococcus faecalis Supplementation as Direct-Fed Microbials on Rumen Microbiota of Boer and Speckled Goat Breeds
Source: Vet Sci. 2021 Jun 7;8(6):103. doi: 10.3390/vetsci8060103 (PMC8229190; doi:10.3390/vetsci8060103)
Supplement: Supplementary file 1 [file vetsci-08-00103-s001.zip › vetsci-1204447-supplementary.pdf]

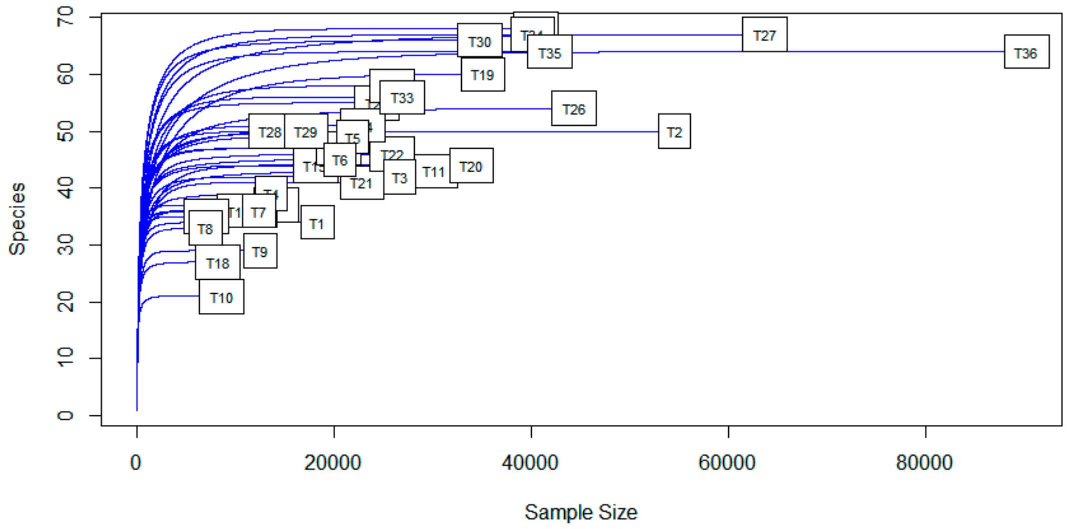

Supplementary Figure S1: Rarefaction curves for rumen microbial communities for each sample, showing species accumulation in the goats. T1-T18 indicates samples collected at the beginning of the trial (day 1) and T19-T36 were collected at the end of the trial (day 30).
